# Traffic Feature Selection and Distributed Denial of Service Attack Detection in Software-Defined Networks Based on Machine Learning

**DOI:** 10.3390/s24134344

**Published:** 2024-07-04

**Authors:** Daoqi Han, Honghui Li, Xueliang Fu, Shuncheng Zhou

**Affiliations:** College of Computer and Information Engineering, Inner Mongolia Agricultural University, Hohhot 010018, China; 15623587331@163.com (D.H.); fuxl_imau@163.com (X.F.); zhousc1121@163.com (S.Z.)

**Keywords:** distributed denial of service (DDoS), feature selection (FS), machine learning (ML), software-defined network (SDN)

## Abstract

As 5G technology becomes more widespread, the significant improvement in network speed and connection density has introduced more challenges to network security. In particular, distributed denial of service (DDoS) attacks have become more frequent and complex in software-defined network (SDN) environments. The complexity and diversity of 5G networks result in a great deal of unnecessary features, which may introduce noise into the detection process of an intrusion detection system (IDS) and reduce the generalization ability of the model. This paper aims to improve the performance of the IDS in 5G networks, especially in terms of detection speed and accuracy. It proposes an innovative feature selection (FS) method to filter out the most representative and distinguishing features from network traffic data to improve the robustness and detection efficiency of the IDS. To confirm the suggested method’s efficacy, this paper uses four common machine learning (ML) models to evaluate the InSDN, CICIDS2017, and CICIDS2018 datasets and conducts real-time DDoS attack detection on the simulation platform. According to experimental results, the suggested FS technique may match 5G network requirements for high speed and high reliability of the IDS while also drastically cutting down on detection time and preserving or improving DDoS detection accuracy.

## 1. Introduction

In today’s digital age, the promotion and application of 5G technology have significantly enhanced the interconnectivity of terminal systems. With numerous connections, low latency, and high speed, 5G technology has considerably aided the rapid development of Internet of Things (IoT) devices and automation in industries. However, this also means that the challenges and threats to cybersecurity have increased accordingly.

An intrusion detection system (IDS), as a key security device or software, plays a particularly important role in 5G networks. The IDS is responsible for monitoring computer network or system activities and detecting and responding to malicious activities, unauthorized access, or abnormal behavior to protect the network from infringement [1].

Distributed denial of service (DDoS) attacks are the most dangerous among all network threats, as DDoS can quickly deplete hardware resources, leading to performance degradation or service termination. Although the high bandwidth and low latency characteristics of the 5G network bring a better experience to users, it also provides more convenient conditions for DDoS attacks. Armed with an abundance of resources and increased operational efficiency, cyber attackers are capable of mounting sophisticated assaults, thereby posing a significant and pervasive threat to the information security of individuals, corporations, and institutions alike.

According to NETSCOUT’s threat intelligence report [2], the frequency of DDoS attacks is rising year by year. In 2021, the total number of DDoS attacks reached 9.75 million. In 2022, it increased to 10.53 million, a year-on-year growth of 8%. In 2022, China endured a staggering 1.1667 million DDoS attacks, representing a significant 11.08% of the global tally. Meanwhile, the United States witnessed a marked escalation, with its share of global attacks soaring to 43.29%.

A software-defined network (SDN) [3], a developing network architecture, increases network flexibility and programmability by separating the control plane from the data plane to meet the needs of 5G networks. This feature not only makes network management and control more centralized but also brings new opportunities for intrusion detection. Nevertheless, it is the very centralized control nature of SDN that renders it more susceptible to DDoS attacks [4]. In particular, the SDN controller is a core component in the network. Once it is faulty or attacked, it will cause the entire SDN network to collapse [5]. Consequently, the establishment of an IDS that is adept at real-time and efficient detection of DDoS attacks within an SDN environment is of paramount importance.

As the number of assault features increases, so does the computational cost of IDS, requiring more memory and CPU resources and increasing attack detection time. Feature selection (FS) is a fundamental strategy for improving the performance of an IDS [6,7]. FS can improve the accuracy of pattern classification for various attack types by removing unnecessary and redundant features and selecting the most appropriate subset of features [8]. In artificial intelligence-based IDSs, a crucial aspect is the identification and adoption of a set of optimal features that can accurately detect the attacks in the dataset, thus significantly improving the detection accuracy of the entire system. Therefore, developing a technique is essential to creating trustworthy IDSs that can efficiently decrease the number of characteristics and speed up detection.

Current research grapples with a multitude of challenges, such as ensuring efficiency in processing vast datasets, enhancing the model’s capacity for generalization, assessing the generalizability of FS techniques, and conducting robust evaluations across diverse datasets and a spectrum of machine learning (ML) and deep learning (DL) models. Additionally, DL models often have high resource consumption due to numerous parameters and use outdated datasets, and research results may be limited to specific datasets. These issues limit the applicability of existing methods in real network environments. Moreover, complex DDoS detection models significantly increase the required computational resources. Furthermore, in the realm of DDoS attack detection, DL techniques encounter a variety of obstacles. These include high computational complexity [9], which is often caused by the complexity of model hybrid architectures; high processing overhead [10], especially when implementing and maintaining these models; and a time-consuming detection process [11], which can be a problem in cybersecurity environments that require fast responses.

This paper proposes an innovative FS methodology, termed XRDI, designed to augment the IDS efficacy in identifying DDoS attacks within SDN environments, while concurrently aiming to curtail the detection time. XRDI integrates four sophisticated feature importance selection algorithms to distill the most salient and pertinent features from the original dataset, leveraging their respective feature importance scores. Following this, four ML models are constructed. Based on these, the XRDI effectiveness is verified through extensive simulation experiments on datasets InSDN, CICIDS2017, and CICIDS2018. Also, it is performed in the SDN simulation environment.

The principal contributions of this paper are delineated as follows:

1. A novel FS method known as XRDI is proposed, which integrates four feature importance selection algorithms. This method adeptly extracts the most salient features from the dataset, culminating in enhanced detection accuracy and a substantial reduction in the time required for the detection process.
2. Four ML models based on the XRDI method are constructed, including decision tree (DT), random forest (RF), support vector machines (SVMs), and logistic regression (LR).
3. Extensive experiments have been conducted on three datasets, i.e., InSDN, CICIDS2017, and CICIDS2018. The experimental results show that the ML models based on XRDI are superior to the conventional DDoS detection ones in terms of detection time, computing cost, and accuracy. Moreover, in order to verify the real-time performance of the proposed XRDI-based ML model, it is deployed in the SDN simulation environment. The average detection time of real-time DDoS attack detection is 352.7098 ms, and the average prediction time is only 0.2304 ms.

The outline of this paper is as follows. Section 1 introduces the research background. Section 2 describes the state-of-the-art studies. The suggested methodology in this paper is thoroughly explained in Section 3. Section 4 describes the conducted experiments and discusses the experimental results. The entire study is finally summarized in Section 5.

## 2. Related Work

In the 5G environment of SDN, the detection of DDoS attacks faces new challenges and opportunities [12,13,14,15,16]. As network traffic becomes increasingly complex, traditional signature-based detection methods are no longer able to meet demand, while anomaly-based detection methods face problems such as high false alarm rates and consumption of computing resources.

Lei et al. [12] proposed an anomaly detection algorithm that combines ensemble learning with the self-attention mechanism. The algorithm performed well in the SDN-based 5G networks. However, its computational complexity is high, which may not be suitable for application scenarios that require extremely high response speeds.

Li et al. [13] developed an intelligent IDS based on ML, which improved the detection performance and reduced the overhead.

In the face of DDoS attacks initiated by malicious organizations and users, the 5G network based on SDN has encountered serious security threats. Alamri et al. [14] proposed a new security scheme by combining an ML algorithm and an adaptive bandwidth mechanism to enhance the security of an SDN controller. Li et al. [15] proposed a two-stage intelligent model by creating self-generated datasets, which can distinguish normal and abnormal traffic and classify 21 kinds of DDoS attacks. However, these studies lack investigations of the adaptability of the model to emerging attack types, as well as the generalization ability to different network environments.

Kim et al. [16] focused on DDoS attacks launched by IoT devices in the 5G core network and effectively improved the detection performance through FS. Nevertheless, the existing literature has yet to explore the FS method’s resilience against a variety of attack modes within dynamic network settings. Additionally, it overlooks the challenge of sustaining high detection precision without compromising on response time.

Although many solutions to detect DDoS attacks in the SDN environment have been proposed, it is still urgent to take effective measures to protect the SDN network. To reach the objectives of ultra-low latency in 5G network services and meet the time required for the real-time detection of large-capacity DDoS attacks in a 5G network environment, FS has emerged as an important research direction.

El Sayed et al. [17] proposed an FS method that combines information gain (IG) and RF algorithms to detect DDoS attacks in SDN environments. This method selects the 10 most relevant features to detect DDoS attacks from 48 potential features. The selected features are then combined with long short-term memory (LSTM) and autoencoder. It was evaluated using the InSDN, CICIDS2017, and CICIDS2018 datasets. The experimental results demonstrated the effectiveness of the proposed FS method. However, the study did not include evaluations using other ML or DL models.

Liu et al. [18] introduced an FS method known as FSM, which is based on the extraction of multiple feature subsets and the fusion of their results. This method optimizes the FS process through the application of a quick, non-dominated sorting algorithm and mutual information. Through experiments conducted on 20 well-known datasets, it has been demonstrated that the FSM is capable of reducing data dimensionality effectively. Moreover, it shows an improvement in classification performance.

Furthermore, a hybrid FS and correlation analysis method was presented by Chanu et al. [19]. Based on this FS technology, the 9 most relevant features for the classification task were finally accurately identified and selected from 78 features in the original dataset. A multi-layer perceptron using a genetic algorithm (MLP-GA) as a classifier was evaluated on the CICIDS2017 dataset. And MLP-GA improved the accuracy and efficiency of DDoS attack detection. Nevertheless, GA-based FS and classification methods may encounter challenges, including the substantial computational expense and the intricacies associated with parameter tuning.

Zhou et al. [20] proposed the SAFE system. It removes highly correlated redundant features. Through the utilization of FS and threshold tuning, 21 unidirectional statistical features were extracted from three categories of network traffic. The Pearson correlation coefficient (PCC) was adopted to assess the dependency between features. A fast and accurate classification of DDoS attack streams was achieved. However, the FS process and threshold adjustment may still need to be optimized for efficiency for large-scale datasets.

Das et al. [21] proposed an integrated framework known as ensemble feature selection (EnFS) that combines seven well-known FS methods and generates 11 optimal feature subsets using majority voting (MV) techniques. These methods include filtered (e.g., Pearson correlation coefficient, chi-square test), wrapper (e.g., recursive feature elimination), and embedded methods (e.g., LASSO regression and RF). With an integrated supervised ML framework, the study classified data from the NSL-KDD dataset with 97.5% accuracy.

Thakkar et al. [22] proposed an FS technology based on statistical importance fusion, which uses the absolute value of the standard deviation, mean, and median difference to select features. The study used a deep neural network (DNN) as a classifier and achieved an accuracy of 99.80%. Despite achieving good results in accuracy, DL models frequently require enormous amounts of training data, which may limit their application in data-constrained situations.

Eldhai et al. [23] proposed a novel approach for both FS and stream classification, which integrated ML and the Boruta FS technique. This approach’s performance was evaluated on both real and synthetic traffic, and high accuracy was attained.

Tripathi et al. [24] introduced a new FS optimization method named “chi-rev”, which is designed to enhance the FS efficiency. The primary goal of this method is to streamline the process and eliminate irrelevant features. The chi-rev method was able to eliminate nearly 51% of the extraneous characteristics regarding the CICIDS2017 dataset. Moreover, by integrating the RF classifier, the method achieved a remarkable detection accuracy of 99.9%. However, further evaluation is required to verify the method across different datasets in terms of robustness and generalizability.

Ayuba et al. [25] proposed a framework for wireless sensor networks based on clustering and variable selection-integrated ML algorithms (CBWSN_VSEMLA) for detecting DoS attacks. It is very effective in wireless sensor networks, but the PCA_RandomForest model has high computational complexity and takes 231.64 s to train. This indicates that the model may not be efficient enough when dealing with large-scale datasets or when real-time detection is required.

Hybrid strategies are widely adopted for FS and classification to detect network assaults [26]. Of particular interest is the fact that these strategies incorporate FS techniques to enhance the IDS performance. Still, the combination of intrusion detection and FS does not lie solely in simply enhancing system performance, but, more importantly, it should focus on improving classification accuracy while reducing attack detection time.

In summary, FS is important in building IDSs. Appropriate FS techniques can not only lower training time and computing costs but also increase the model’s performance and efficiency. Further, they can improve the model’s capacity to detect intrusion activities. Table 1 presents comparisons of the studies mentioned above.

## 3. Proposed DDoS Intrusion Detection Method

**Figure 1:**
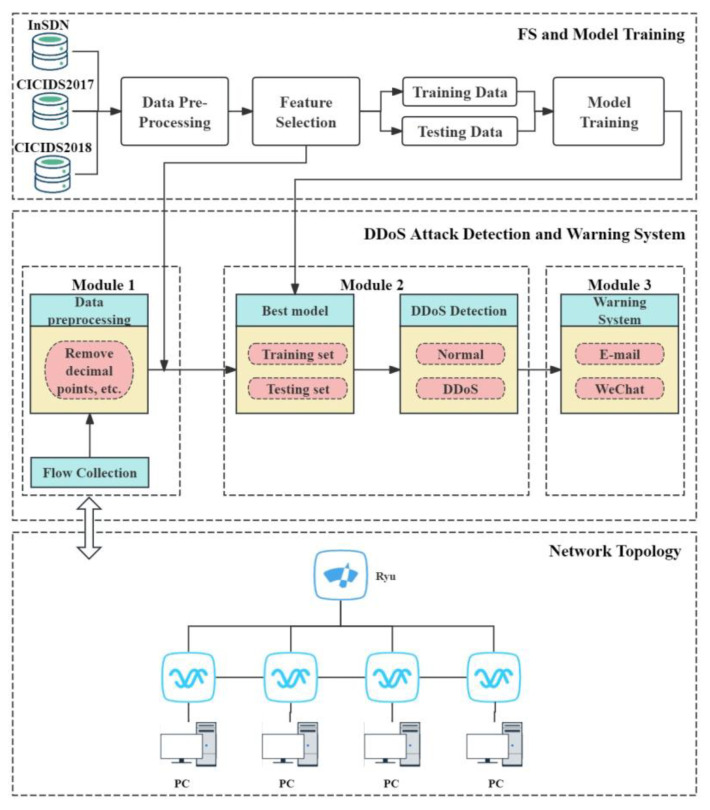
Architecture of the proposed approach.

The proposed architecture of the IDS detection solution in the SDN network is depicted in Figure 1. This architecture is composed of three parts, i.e., an FS and model training part, a DDoS attack detection and warning system, and an SDN network.

The first part focuses on FS and model training. Three different datasets are employed for DDoS attack detection, including InSDN, CICIDS2017, and CICIDS2018. After data preprocessing, the proposed FS method is exploited to achieve feature subsets from the above three datasets. Then, each data subset is divided into the training set and the testing set. On top of this, four detection models are constructed based on four machine learning models, i.e., DT, SVM, RF, and LR. In the end, the best-performing model is selected from these four models and acts as the classifier for DDoS attack detection in SDN-based 5G networks.

The FS method specifically considers the characteristics of 5G networks, such as high bandwidth, low latency, and massive connectivity, to ensure the model effectively addresses DDoS attacks in a 5G environment.

The second part involves DDoS attack detection and alerting, which comprises three modules. Module one first collects traffic from the SDN controller Ryu and then preprocesses the traffic data for FS, with a particular focus on the traffic patterns and characteristics of 5G networks. After FS, the second module employs the best model among the above four models for real-time DDoS attack detection, leveraging the low latency of 5G networks for rapid detection. When a DDoS attack is detected, the third module sends email and WeChat notifications to network administrators for a timely response. Thus, it can ensure the high reliability and security of 5G networks.

The third part is the SDN simulation platform, which includes the Ryu controller and a simulated network topology built on Mininet. This platform is employed to verify the efficiency of the proposed detection system in a simulated 5G network environment.

### 3.1. Dataset Selection

This section introduces the datasets and their features exploited for FS and model training in this paper.

#### 3.1.1. Dataset Introduction

High-quality traffic datasets are crucial to building effective anomaly detection systems. However, it is not easy to achieve such datasets. Since the objective is to identify attacks in SDN networks, three datasets, InSDN [27], CICIDS2017 [28], and CICIDS2018 [28] datasets, are utilized in this paper. Among them, InSDN is exploited to train XRDI-based ML models, and the others are used to evaluate the ML model performance.

These three datasets are all CSV files containing multiple total categories. Each dataset contains more than 80 network flow characteristics. As the objective is to detect DDoS attacks, normal traffic and DDoS attack traffic in these three datasets are extracted and labeled as normal and DDoS, respectively. Regarding the CICIDS2017 and CICIDS2018 datasets, the files are employed between Friday, 7 July 2017, and Wednesday, 21 February 2018. The following is a synopsis of each dataset.

1. InSDN

The InSDN dataset was created in 2020 with the goal of solving the deficiencies of prior datasets in identifying attacks in SDN systems. There are overall 343,889 data instances and 84 features per instance. These instances are divided into eight different traffic categories. The dataset contains a variety of attack scenarios, such as loris attacks, SYN flooding, and flooding of TCP, UDP, and ICMP. Among these traffic instances, 68,424 instances belong to regular traffic, and 275,465 instances belong to attack traffic, among which there are 121,942 instances of DDoS attack traffic.

2. CICIDS2017

The CICIDS2017 dataset was proposed by the Canadian Institute for Cyber Security (CIC) in 2017. It contains five days of network traffic generated between Monday, July 3, and Friday, 7 July 2017. This dataset contains the necessary instances of 11 common updated attacks and 85 features per instance, such as DoS, DDoS, brute force, XSS, SQL injection, penetration, port scanning, and botnet.

3. CICIDS2018

The CICIDS2018 dataset was generated based on a collaborative project between the CIC and the Communications Security Establishment (CSE). This dataset is an extended version of the CICIDS2017 dataset. All traffic data were collected within 10 days, with a total number of 16,233,002 instances and 80 features per instance, of which the attack instance size accounts for 17% of the entire data. The CICIDS2018 dataset covers a wide variety of network traffic scenarios, including normal activities and various types of network attacks.

#### 3.1.2. Feature Description

In SDN networks, the characteristics of data traffic are crucial for network management and security. Earlier studies mostly used 50 feature subsets [29] and 48 feature subsets [17] as research targets. Different from this, this paper retains all features with feature values of 0 in the three datasets and only deletes the source IP, source port, flow ID, and timestamp features. Experiments are conducted based on the remaining 77 features, and the resulting 77 feature subsets are shown in Table 2.

### 3.2. Data Preprocessing

The following steps are adopted to fulfill dataset preprocessing aiming to enhance the ML model’s stability and performance.

1. Data cleaning

There are plenty of infinite and missing (NaN) values in the CICIDS2017 and CICIDS2018 datasets. Since both datasets have enough instances, those instances were simply deleted with missing values or infinite values.

2. Numerical labeling

ML/DL models require the numerical input data to facilitate model training and result interpretation. This paper is dedicated to the classification of normal and DDoS attack traffic from various instances. In this context, normal traffic is assigned the label ‘0’, while DDoS attack traffic is designated the label ‘1’.

3. Dataset balancing

Table 3 illustrates the disparity in the distribution of benign and DDoS attack traffic across the three datasets, with a significant variation in the number of samples. This imbalance may lead the model to favor predictions of the predominant category. Achieving a balanced dataset allows the model to handle each category with greater equity, thereby enhancing the predictive accuracy for the underrepresented classes.

Recognizing the limited quantity of normal traffic samples in both the InSDN and CICIDS2017 datasets, this paper employs a technique known as the synthetic minority over-sampling technique (SMOTE) [28] to augment the dataset with an expanded array of normal traffic instances. This method effectively balances the class imbalance problem in the dataset by synthesizing new samples. In contrast, the CICIDS2018 dataset boasts a substantial sample size, with an abundance of attack traffic samples. This paper opts for a random under-sampling technique [29] to curtail the volume of attack traffic instances, thereby achieving a more balanced and representative dataset. By employing this strategy, the paper endeavors to refine the dataset’s sample distribution, thereby enhancing the efficacy of model training and bolstering the precision of predictions.

Table 4 presents the distribution of the three datasets after the balancing process has been applied, showcasing a more equitable representation of samples across different categories.

4. Data normalization

This paper employs the min–max normalization technique [30], a method that rescales each feature’s minimum value to 0, maximum value to 1, and all other values to a proportional decimal between these bounds. This approach is essential due to the varying magnitudes of the data features. The transformation function is formulated as Equation (1):

(1) $$F_{new}=\frac{F-F_{min}}{F_{max}-F_{min}}$$

where *F* denotes the feature value to be scaled down, $F_{max}$ is the maximum value, and $F_{min}$ is the minimum value for a particular feature in the SDN network traffic.

### 3.3. Proposed FS Method XRDI

**Figure 2:**
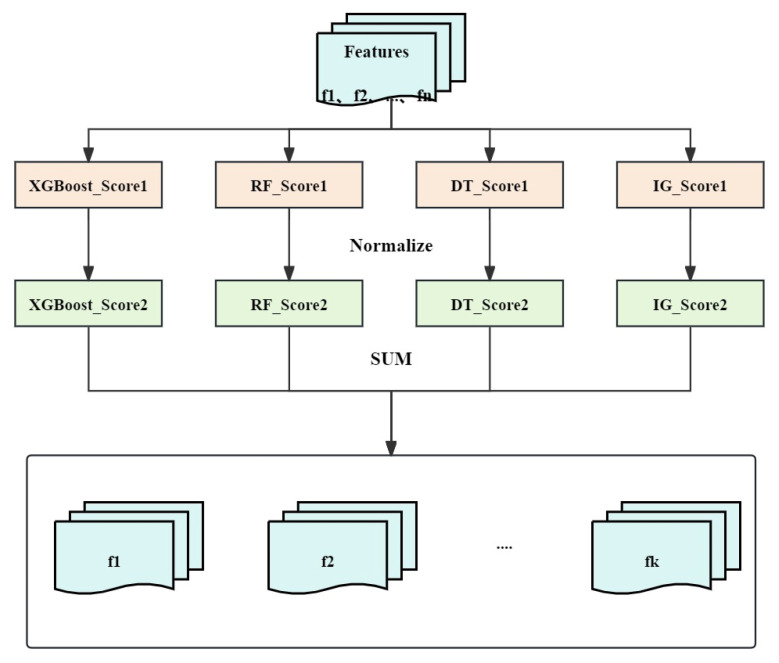
Flowchart of the XRDI method.

This section describes the proposed FS method XRDI in detail for identifying each dataset’s most representative features. Figure 2 illustrates the XRDI flowchart.

This paper presents a novel FS method named XRDI aimed at amplifying the IDS effectiveness and precision while also saving time. XRDI is based on feature importance scores to extract relevant features from intrusion detection datasets. And the feature importance scores are calculated through integrations of four ML algorithms, i.e., extreme gradient boosting (XGBoost), RF, DT, and IG. When conducting an analysis of feature importance, these four algorithms are employed to thoroughly assess the impact of each individual feature within the dataset.

The XGBoost algorithm calculates feature importance scores by evaluating the feature frequency that appeared in all gradient boosting decision trees. As 5G network traffic characteristics change rapidly, timely and accurate feature extraction is crucial. XGBoost is adept at efficiently managing rapid and frequent data fluctuations, swiftly pinpointing significant features.

The RF algorithm is also exploited to evaluate feature importance comprehensively. RF integrates scores from multiple DTs. And the traditional DT algorithm is employed to further verify feature importance, intuitively demonstrating the contribution of features to model decision-making. Moreover, 5G networks support diverse devices and high mobility, and RF and DT are well-suited for adapting to complex traffic characteristics.

The IG algorithm is exploited to calculate feature importance scores as well, which evaluates features based on their contribution to data classification results. In 5G networks, network slicing technology requires distinguishing traffic characteristics between different slices. This can be effectively handled by the IG algorithm.

The advantage of the XRDI method is that it can comprehensively capture the contribution of features to the intrusion detection task, thereby improving the FS accuracy of FS and the IDS performance. Through this comprehensive evaluation, XRDI can identify the most effective features under different network environments and diverse intrusion behaviors, enhancing the adaptability and robustness of the system.

The input to the XRDI method is a dataset containing n features. Let $f_{i}$ represent the *i*-th feature in the dataset, *i* = 1, 2, 3, …, n. The steps of the XRDI method are elaborated in detail as follows.

(1) Calculate XGBoost_Score1. For each feature f in the dataset, XGBoost_Score1 refers to its feature importance weight in XGBoost [31], which is calculated by Formula (2):

(2) $$weight\left(f\right)=\sum_{i=1}^{N}Gain\left(f,i\right)\times Coverage\left(f,i\right)$$

where, for the feature f, weight(f) represents its importance weight, Gain(f,i) its gain at the *i*-th node split, Coverage(f,i) its coverage at the *i*-th node, and N the total number of nodes.

(2) Calculate XGBoost_Score2. In order to render the feature importance scores across various models comparable, Equation (1) is employed to normalize XGBoost_Score1, and the normalized feature importance score XGBoost_Score2 is achieved.
(3) Calculate RF_Score1. RF_Score1 refers to the feature importance in the RF model [32], which is calculated using Formula (3) for each feature f in the dataset.

(3) $$Importance\left(f\right)=\frac{1}{N}\sum_{t}IMP\left(f,t\right)$$

where N represents the total number of trees, t is the index of the tree, and IMP(f,t) is the Gini impurity reduction of the feature f in the tree t.

For each feature f, its Gini impurity reduction IMP(f,t) on all nodes of all trees is averaged to obtain the importance score of feature f.

Assuming that a feature f is split on the node of the tree t, IMP(f,t) is calculated using Formula (4).

(4) $$IMP\left(f,t\right)=GINI\left(t\right)-{GINI}_{split}\left(t\right)$$

where GINI(t) is the Gini impurity of the node before splitting, and ${GINI}_{split}\left(t\right)$ is the Gini impurity of the node after splitting.

(4) Calculate RF_Score2.RF_Score1 is normalized with Equation (1) to obtain RF_Score2.
(5) Calculate DT_Score1 and DT_Score2. The DT algorithm is pursued to calculate the feature importance score, known as DT_Score1. By applying Equation (1), the normalized feature importance score DT_Score2 is achieved.
(6) Calculate IG_Score1 and IG_Score2. The information gain IG(X,Y) is an indicator used to measure the importance of a feature for classification tasks, that is, the entropy of the target variable Y minus the weighted sum of the conditional entropy of the feature X. It is calculated as follows:

(5) $$IG\left(X,Y\right)=Entropy\left(Y\right)-\sum_{i=1}^{n}\frac{\left|X_{i}\right|}{\left|X\right|}\times Entropy\left(Y|X_{i}\right)$$

where IG(X,Y) represents the information gain of feature X concerning the target variable Y. Entropy(Y) denotes the entropy of the target variable Y. |X| indicates the total number of samples, and $\left|X_{i}\right|$ represents the number of samples corresponding to the ith value of feature X. $Entropy\left(Y|X_{i}\right)$ denotes the conditional entropy of the target variable Y given that the feature X takes the value $\left|X_{i}\right|$.

The IG method is also used to calculate the feature importance score, and this article names its original score IG_Score1.

Similarly, after the normalization processing of Equation (1), IG_Score2 can be obtained.

(7) Calculate CombinedFeatureScore. The normalized feature importance scores of the above four models are summed to obtain a combined feature importance score, expressed as CombinedFeatureScore=XGBoost_Score2+RF_Score2+DT_Score2+IG_Score2.
(8) Sort features by score. All features in the dataset are sorted according to their combined feature importance score. Finally, those features are identified that are most critical to enhance model performance based on their ranks.

#### FS Results

To clarify the difference between the features selected by XRDI and the features selected by the basic methods, this section first uses four basic FS methods to select features on the three datasets mentioned above.

The features selected by the four basic FS methods and XRDI are shown in Table 5 from the three datasets, where the third column lists the selected feature index.

It can be observed that, although the categories and numbers of features on the three datasets are the same, the features selected by each FS method on different datasets are different. For each dataset, the selected subset of features varies with the FS methods. As far as XRDI is concerned, there are four common features (i.e., No. 37, 44, 50, and 76) between CICIDS2017 and CICIDS2018, only two common features (No. 8 and 37) between InSDN and CICIDS2017, and only two common features (No. 37 and 74) between InSDN and CICIDS2018. Table 6 elaborates on these six common features.

**Figure 3:**
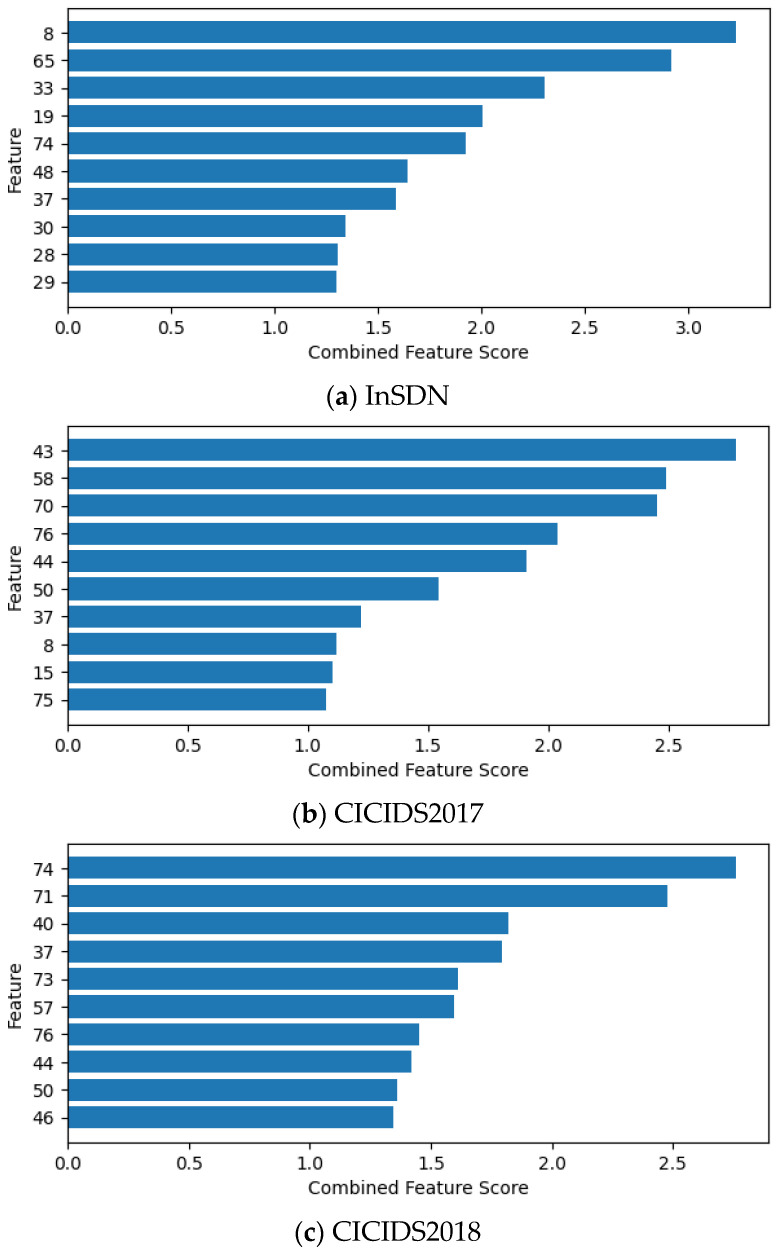
Feature subsets and their CombinedFeatureScore selected by XRDI method.

When the XRDI method is applied to select features on the InSDN, CICIDS2017, and CICIDS2018 datasets, it first calculates the importance score of each feature. Then, it ranks the features according to this score. Figure 3a–c show the top 10 features on each dataset, where the *X*-axis represents the CombinedFeatureScore and the *Y*-axis represents the serial number of the feature.

As can be seen from Figure 3, the CombinedFeatureScore for the last three features remained roughly the same. Combined with the literature [17], this paper selects the top 10 feature subsets for evaluation. The specific features selected by the XRDI method are shown in Table 7.

Taking the InSDN dataset as an example, ten features selected are important in detecting DDoS attacks in SDN networks. First, the No. 8 (Bwd Header Len) and No. 37 (Fwd Header Len) features provide information about the packet and flow structure, helping to identify abnormal traffic patterns. In 5G networks, the high bandwidth and low latency characteristics cause traffic features to change more rapidly, making these features crucial for timely detection and response to DDoS attacks. Feature No. 19 (Bwd Pkts/s), No. 33 (Flow Pkts/s), and No. 48 (Fwd Pkts/s) provide information about the flow rate. Abnormally high rates may indicate DDoS attacks. In the 5G environment, the diversity of device types and higher mobility result in more complex traffic patterns, and these flow rate features can help identify abnormal behavior. The No. 28 (Flow Duration) and No. 74 (Tot Fwd Pkts) features are helpful in detecting an abnormal number of packets and flow duration, while No. 29 (Flow IAT Max) and No. 30 (Flow IAT Mean) provide information about the interval time of the flow, helping to discover abnormal interval time patterns. In 5G networks, network slicing technology allows different services to share the same physical network while maintaining independent virtual networks, necessitating these features to distinguish traffic characteristics between different slices. The No. 65 (Protocol) feature indicates the type of protocol that may be exploited by the attack. Comprehensive analysis of these features can effectively help the identification of and response to DDoS attacks in 5G networks.

### 3.4. Split the Dataset

This paper uses the test_train_split function from the Sklearn package to split datasets. Each dataset is split into a 7:3 ratio, which indicates that 70% of the dataset is utilized for training and the remaining 30% is used for model testing to ensure prediction accuracy.

### 3.5. ML Model Training

In the DDoS attack detection stage, this paper uses four different ML algorithms to build classifiers, i.e., DT, SVM, RF, and LR.

1. The DT model is adept at managing feature interactions and thus is often favored in contexts requiring high comprehension.
2. The RF model combines the benefits of several decision trees while reducing the model’s uncertainty and overfitting risk via ensemble learning. In RF, each decision tree is trained on a random subset of the dataset, which increases the overall model’s variety and, as a result, predictability. Furthermore, each feature’s Gini index is calculated to determine its importance in random forests.
3. SVM is a strong supervised learning technique that is frequently used for classification and regression applications. SVM aims to discover an appropriate hyperplane in the feature space to differentiate between categories. The kernel trick is a critical component of SVM, allowing the technique to effectively handle nonlinear issues in high-dimensional space. The kernel function allows SVM to calculate the inner product of data points in high-dimensional space without explicitly mapping them to that space.
4. The LR model’s basic linear relationship makes it easy to interpret.

By thoroughly analyzing the performance of these four ML models on the test set, the best-performing model is chosen as a classifier to improve the accuracy and efficiency of DDoS intrusion detection while also reducing the detection time of DDoS attacks.

## 4. Experimental Design and Result Analysis

To confirm the suggested method’s efficacy, a large number of experiments were conducted. In addition to the tests on three public sets, the performance of the proposed method was also verified in the SDN simulation environment and compared with several existing research works. The experimental results clearly show the advantages and possible application value of the proposed approach.

### 4.1. Experimental Environment

This article uses the Scikit-learn library in the Python programming language to evaluate the performance of the DDoS attack detection model. The hardware and software parameters of the experimental bench are shown in Table 8.

### 4.2. Evaluation Indicators

The model’s performance is measured using the most common metrics, including *accuracy*, *precision*, *recall*, and *F1-score*.

*Accuracy*: The computation involves dividing the total number of accurate forecasts by the total number of forecasts produced. The formula for calculating it is given below:

(6) $$Accuracy=\frac{TP+TN}{TP+TN+FP+FN}$$

where true positive (*TP*) denotes an attack packet that is correctly categorized as an attack, false positive (*FP*) denotes a benign packet that is incorrectly categorized as an attack, true negative (*TN*) denotes a benign packet that is correctly categorized as normal, and false negative (*FN*) denotes an attack packet that is incorrectly categorized as normal, and these metrics were computed based on the confusion matrix.

*Precision*: It is a measure of the proportion of true positives among all positive predictions and is used to determine how much attack traffic is correctly identified. The formula is calculated as follows:

(7) $$Precision=\frac{TP}{TP+FP}$$

*Recall*: *Recall* quantifies the percentage of correctly predicted actual positives and measures the expected attack rate relative to the overall attack traffic, with recall illustrating its ability to capture all actual positives. The formula is calculated as follows:

(8) $$Recall=\frac{TP}{TP+FN}$$

*F1-score*: This statistic allows for a balanced evaluation of the model’s performance by balancing precision and recall. It is computed as the average of precision and recall that has been reconciled. The formula is calculated as follows:

(9) $$F1-score=2\times \frac{Precision\times Recall}{Precision+Recall}$$

### 4.3. Experimental Results and Analysis

Table 9 shows the *accuracy* (Acc, %), *precision* (Pre, %), *recall* (Rec, %), and *F1-score* (F1, %) with different FS methods on the InSDN, CICIDS2017, and CICIDS2018 datasets. When all 77 features of the datasets are employed for classification during model training, the accuracy rates on the three datasets are the highest, which are 99.98%, 99.97%, and 100%, respectively.

After 10 features are extracted by XGBoost, RF, DT, and IG FS algorithms each, four classification models are then built based on these features accordingly. Table 9 shows that their classification accuracy dropped slightly, no more than 0.1%. It can be observed that the 10 features selected using the DT FS method have the highest accuracy on the three datasets when using the DT classification algorithm, while the 10 features selected using the RF FS method have the lowest accuracy on the three datasets when using the LR classification algorithm.

DT and RF achieved the highest accuracy, reaching 99.97% when trained on four classification models sampled from the InSDN dataset using 10 features selected by the proposed XRDI FS algorithm. On the three datasets, the overall performance of DT and RF was higher than that of SVM and LR.

**Figure 4:**
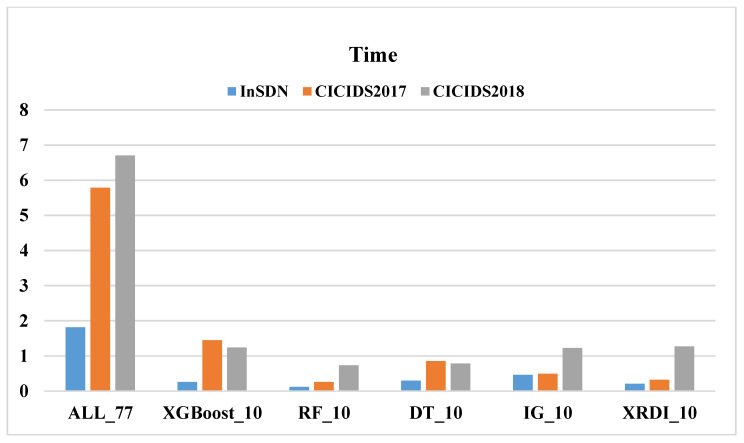
The time cost of using the DT algorithm on the three datasets.

**Figure 5:**
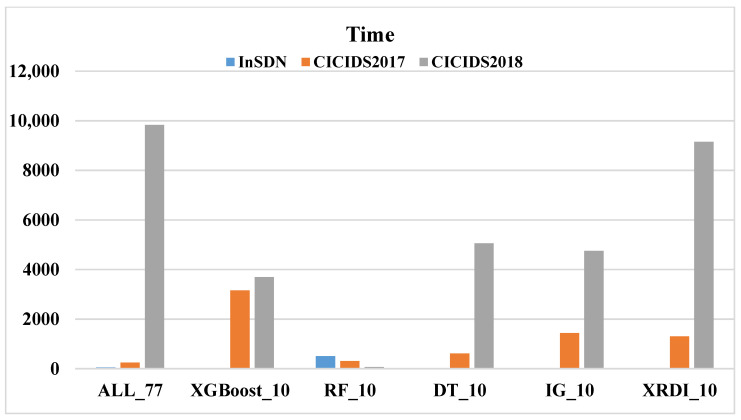
The time cost of using the SVM algorithm on three datasets.

**Figure 6:**
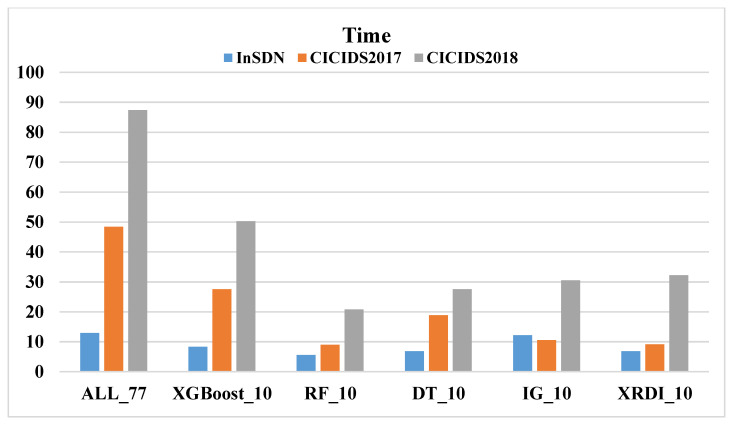
The time cost of using the RF algorithm on three datasets.

**Figure 7:**
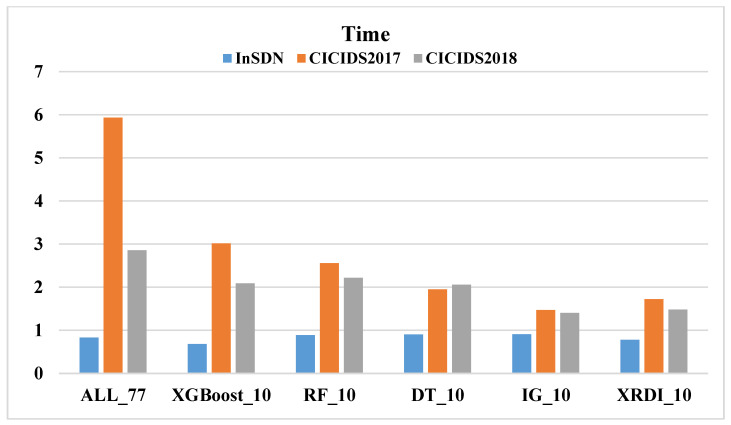
The time cost of using the LR algorithm on three datasets.

Similarly, this paper uses different FS methods on three datasets, adopts four ML models, and compares the time cost (the sum of detection time and prediction time). Figure 4, Figure 5, Figure 6 and Figure 7 shows the time costs of using four ML algorithms on three datasets. When all 77 features are used for classification, the time costs on the three datasets are as high as 1.8144 s, 5.7898 s, and 6.7074 s. As the number of features decreases, the time cost also gradually decreases; when using the 10 features selected by the XRDI method for classification, the time costs are 0.208, 0.3228, and 1.2745 s, which are slightly higher than those using the RF classification algorithm. When using the 10 features selected by the XRDI method for classification, the time costs are 0.208 s, 0.3228 s, and 1.2745 s, which are slightly higher than the time cost of using the RF classification algorithm; when using the SVM algorithm for classification, since SVM has multiple parameters that need to be tuned, such as regularization parameters (C), kernel function parameters, etc., different parameter combinations lead to different levels of model performance. When using the RF and LR classification algorithms, the time cost also decreases as the number of features decreases (the time costs are both higher than DT), but when the RF algorithm is used, the time cost on the CICIDS2018 dataset increases.

Figure 7 illustrates the time cost of using the LR algorithm across three datasets. For the CICIDS2017 dataset, as the number of features decreases from 77 to 10, the time cost gradually reduces. The shortest time of 1.4695 s is achieved with 10 features selected using the IG FS algorithm. When the XRDI FS method is employed, the time cost slightly increases to 1.7222 s. This trend is similarly observed in the CICIDS2018 dataset. The most notable result is for the CICIDS2017 dataset, where the detection time reaches 5.9336 s when using all 77 features. By applying the proposed XRDI FS method, the number of features is reduced by 87.01%, and the time cost decreases by 70.97%.

In summary, the use of the XRDI FS method has achieved a significant reduction in the number of features on the three datasets, reducing the data dimension while maintaining model accuracy. Taking the DT classification algorithm as an example, compared with using all features, the number of features is reduced by 87.01% and the time cost is reduced by 88.53%, which means that model training and prediction can be performed faster while maintaining model performance. In general, the application of the XRDI FS method and DT not only improves the operating efficiency of the model but also reduces the computational cost and effectively shortens the time of model training and prediction while maintaining the accuracy of the model. This is crucial for real-time IDS detection systems.

### 4.4. DDoS Attack Detection and Alert Notification

This section introduces the alert system on the SDN simulation platform. As a core component of 5G communication technology, SDNs revolutionize the network architecture by decoupling the control layer and the data layer of the network. Under this architecture, network devices in the data layer are mainly responsible for data transmission while performing operations based on routing commands issued by the control layer. The control layer plays the role of the brain, responsible for formulating policies and routing decisions.

Further, integrating ML techniques into the control layer of the SDN can greatly enhance the intelligence of the network. The control layer can deploy ML models to perform traffic inspection tasks. These intelligent models can analyze network traffic, identify patterns, and make decisions based on real-time data.

**Figure 8:**
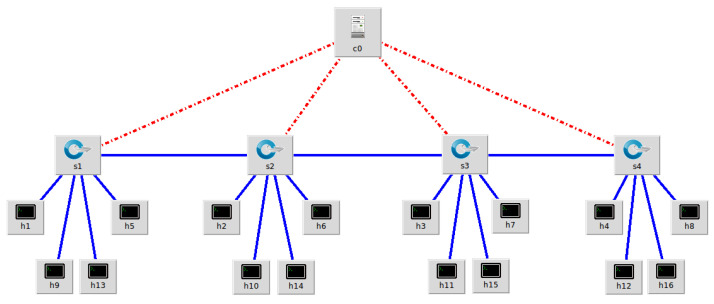
Network topology.

The network topology for this experiment, which consists of sixteen hosts, four Open vSwitch (OVS) switches, and one Ryu controller, is depicted in Figure 8. Host h1 is the victim, while hosts h2 and h3 send normal traffic (ping and file download traffic) to h1. Host h4 is the attacker, simulating a DDoS attack using hping3. The SDN controller is responsible for monitoring the state changes of the switches, requesting and processing traffic statistics, and recording them into a CSV file.

**Figure 9:**
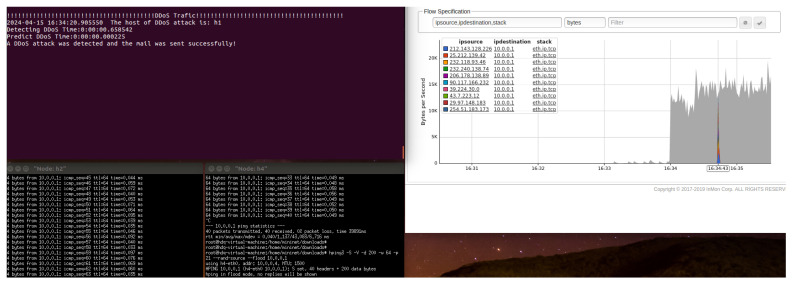
Host h4 uses hping3 to conduct a DDoS attack against host h1.

**Figure 10:**
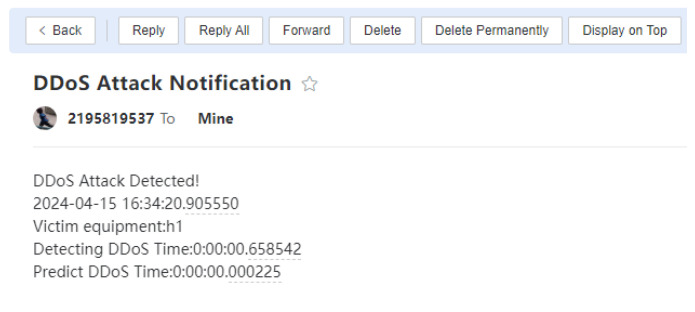
When a DDoS assault is identified, emails are delivered as alerts.

**Figure 11:**
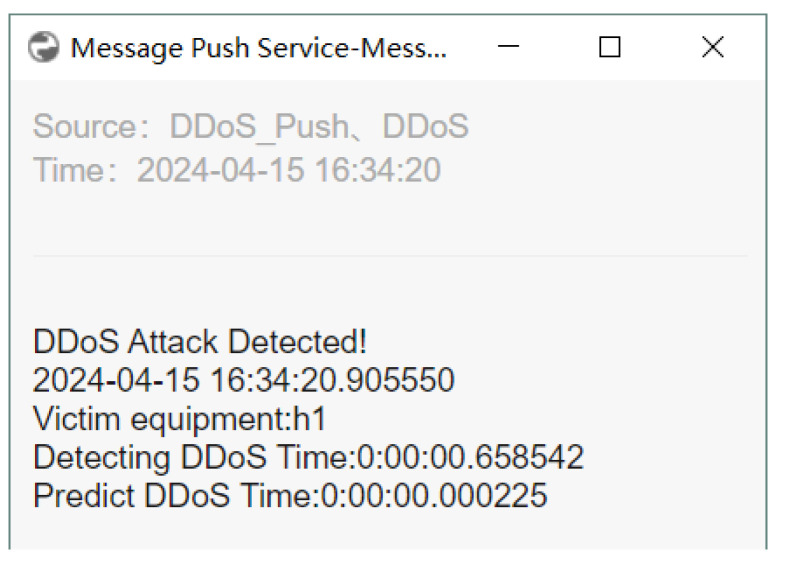
When a DDoS assault is identified, WeChat sends out alarm alerts.

The switches perform data forwarding and collect traffic statistics according to the SDN controller’s instructions. Subsequently, the collected traffic is preprocessed, and FS is performed using the best-performing DT classifier for DDoS attack detection. If the traffic is identified as a DDoS attack, an alert is generated, and email and WeChat notifications are sent; otherwise, the traffic is forwarded normally. As shown in Figure 9, the hping3 tool on host h4 generates random source IP addresses to launch a DDoS attack on host h1. As illustrated in Figure 10 and Figure 11, the warning system notifies the network administrator when an attack is discovered.

The statistical results of ten experiments in the simulation platform are shown in Table 10. The average detection time is 352.7098 ms, while the average prediction time is only 0.2304 ms. This shows the efficacy of the proposed method.

### 4.5. Comparison with Existing Studies

To further verify the improvement and efficacy of the proposed method, this paper conducted comparative experiments with existing representative previous works, including V. Hnamt et al. [10], A. M. Eldhai et al. [23], G. Tripathi et al. [24], J. Chen et al. [33], Z. Liu et al. [34], S. Pande et al. [35], and S. Y. Gündüz et al. [36]. For detailed results of the relevant comparative analysis, please refer to Table 11.

It is particularly worth mentioning that compared with the 12 features used in the literature [33], the method in this paper achieves a significant improvement of 2.75% in accuracy. Compared with the literature [36], even when one feature is reduced, the accuracy is still improved by 4.58%. In addition, the literature [24] proposed the chi-rev FS method, which was assessed using the CICIDS2017 dataset. The number of features was reduced from 79 to 40. When using DT as a classifier, the accuracy, precision, recall, and F1-score were 99.88%, 99.80%, 99.84%, and 99.82%, respectively, while this paper uses the same dataset and the same classifier to achieve better performance. A better binary gray wolf optimization technique for FS was proposed in the literature [34]. From the CICIDS2018 dataset, 26 features were chosen, and SVM, RF, DT, XGBoost, and KNN classifiers were used for evaluation. The results showed that the RF algorithm has the best performance, with an accuracy, precision, recall, and F1-score of 99.13%, 98.43%, 99.92%, and 99.13%, respectively. The performance of the FS method proposed in this article is higher than that in the literature.

These outcomes not only confirm that the FS approach presented in this article can maintain or increase IDS detection accuracy while reducing the number of features but also demonstrate the usefulness and feasibility of the proposed method.

## 5. Conclusions and Future Work

This paper introduces an FS method based on feature importance scoring, along with a real-time DDoS attack detection and alarm system within an SDN environment. These innovations aim to enhance the performance of IDSs and reduce the time required to detect attacks. This method effectively reduces model deviation and bolsters the robustness and reliability of the detection model. Experiments conducted on three datasets—InSDN, CICIDS2017, and CICIDS2018—demonstrate that the proposed method surpasses existing feature sets in terms of accuracy, precision, recall, F1-score, and time cost. The chosen method maintains or improves detection accuracy even when the number of features is reduced. Furthermore, real-time DDoS detection and alarm testing on the Mininet simulation platform further confirmed the effectiveness of this method.

In the future, the proposed XRDI FS method will need to be enhanced to effectively extract features related to other types of attacks. With these improvements, ML models will be capable of identifying a variety of network attacks, including WEB attacks and SQL injections. As for DDoS attacks, the XRDI method also requires further enhancement to accurately identify different subtypes, such as SYN Flood and UDP Flood. Moreover, we plan to collect data in a real-world SDN-based 5G network environment and deploy the proposed model to verify its efficiency in managing real-time network traffic.

## Figures and Tables

**Table 1:** Comparisons of the relevant studies.

Ref.	Dataset	FS Methods	Class Method	Limitations
[13]	KDD Cup 1999	RF	k-means++ AdaBoost	Challenges or limitations that the system may encounter in actual deployment.
[16]	Kitsune GTP-U	Feature importance RFE, RFECV, SFS	DT, RF, KNN, stacking ensemble	The application of FS method in diversified attack modes is not discussed.
[17]	InSDN CICIDS2017 CICIDS2018	RF IG	Autoencoder + RNN	The RNN model consumes more resources.
[18]	20 well-known datasets	Mutual information and the fast non-dominated method	FSM	Users need to adjust this parameter to find the μ value that is most suitable for a specific dataset.
[19]	CICIDS2017	A voting-based hybrid FS technique	MLP-GA	There is no explicit discussion of the model’s performance in a real-time network environment.
[20]	SYN Low Rate Mirai	DDoS attack flow classification system SAFE	Guard, MIR KNN, DP SVM	Using datasets is outdated.
[21]	NSL-KDD	Ensemble feature selection framework (EnFS)	SVM, NB DT, NN LR	The research results may be limited to the specific NSL-KDD dataset.
[22]	NSL-KDD UNSW-NB-15 CICIDS2017	Statistical importance fusion	DNN	May introduce redundant features.
[24]	CICIDS2017	CHI-REV	RF, SVM NB, DT	The generalization ability of this method needs to be further verified on other types of datasets.

**Table 2:** A subset of 77 features in SDN.

No.	Feature Name	No.	Feature Name	No.	Feature Name
1	ACK Flag Cnt	27	Flow Byts/s	53	Idle Max
2	Active Max	28	Flow Duration	54	Idle Mean
3	Active Mean	29	Flow IAT Max	55	Idle Min
4	Active Min	30	Flow IAT Mean	56	Idle Std
5	Active Std	31	Flow IAT Min	57	Init Bwd Win Byts
6	Bwd Blk Rate Avg	32	Flow IAT Std	58	Init Fwd Win Byts
7	Bwd Byts/b Avg	33	Flow Pkts/s	59	Pkt Len Max
8	Bwd Header Len	34	Fwd Act Data Pkts	60	Pkt Len Mean
9	Bwd IAT Max	35	Fwd Blk Rate Avg	61	Pkt Len Min
10	Bwd IAT Mean	36	Fwd Byts/b Avg	62	Pkt Len Std
11	Bwd IAT Min	37	Fwd Header Len	63	Pkt Len Var
12	Bwd IAT Std	38	Fwd IAT Max	64	Pkt Size Avg
13	Bwd IAT Tot	39	Fwd IAT Mean	65	Protocol
14	Bwd Pkt Len Max	40	Fwd IAT Min	66	PSH Flag Cnt
15	Bwd Pkt Len Mean	41	Fwd IAT Std	67	RST Flag Cnt
16	Bwd Pkt Len Min	42	Fwd IAT Tot	68	Subflow Bwd Byts
17	Bwd Pkt Len Std	43	Fwd Pkt Len Max	69	Subflow Bwd Pkts
18	Bwd Pkts/b Avg	44	Fwd Pkt Len Mean	70	Subflow Fwd Byts
19	Bwd Pkts/s	45	Fwd Pkt Len Min	71	Subflow Fwd Pkts
20	Bwd PSH Flags	46	Fwd Pkt Len Std	72	SYN Flag Cnt
21	Bwd Seg Size Avg	47	Fwd Pkts/b Avg	73	Tot Bwd Pkts
22	Bwd URG Flags	48	Fwd Pkts/s	74	Tot Fwd Pkts
23	CWE Flag Count	49	Fwd PSH Flags	75	TotLen Bwd Pkts
24	Down/Up Ratio	50	Fwd Seg Size Avg	76	TotLen Fwd Pkts
25	ECE Flag Cnt	51	Fwd Seg Size Min	77	URG Flag Cnt
26	FIN Flag Cnt	52	Fwd URG Flags		

**Table 3:** Original sample counts of traffic types in the InSDN, CICIDS2017, and CICIDS2018 datasets.

Dataset	0	1
InSDN	68,424	121,942
CICIDS2017	97,686	128,025
CICIDS2018	360,833	687,742

**Table 4:** Post-balancing sample counts of traffic types in the InSDN, CICIDS2017, and CICIDS2018 datasets.

Dataset	0	1
InSDN	100,000	121,942
CICIDS2017	128,025	128,025
CICIDS2018	360,833	360,833

**Table 5:** Feature subsets are selected based on four FS algorithms.

FS Method	Dataset	Feature No. Selected
XGBoost	InSDN	8, 19, 33, 37, 59, 60, 64, 65, 71, 74
	CICIDS2017	19, 27, 28, 31, 38, 40, 4857, 58, 76
	CICIDS2018	30, 31, 32, 40, 41, 48, 57, 58, 73, 74
RF	InSDN	8, 19, 33, 37, 59, 60, 64, 65, 71, 74
	CICIDS2017	34, 37, 43, 44, 50, 58, 70, 71, 74, 76
	CICIDS2018	11, 37, 43, 44, 46, 50, 70, 71, 74, 76
DT	InSDN	4, 8, 19, 24, 30, 31, 41, 59, 65, 72
	CICIDS2017	19, 21, 31, 32, 39, 43, 57, 58, 70, 76
	CICIDS2018	28, 29, 37, 58, 65, 71, 73, 74, 75, 76
IG	InSDN	8, 9, 10, 13, 19, 28, 29, 30, 33, 65
	CICIDS2017	14, 15, 21, 37, 58, 64, 68, 70, 75, 76
	CICIDS2018	8, 37, 40, 43, 44, 46, 50, 57, 69, 73
XRDI	InSDN	8, 19, 28, 29, 30, 33, 37, 48, 65, 74
	CICIDS2017	8, 15, 37, 43, 44, 50, 58, 70, 75, 76
	CICIDS2018	37, 40, 44, 46, 50, 57, 71, 73, 74, 76

**Table 6:** Elaboration of the common features selected from InSDN, CICIDS2017, and CICIDS2018 datasets.

No.	Feature Name	Feature Description
8	Bwd Header Len	The total bytes used for headers in the backward direction
37	Fwd Header Len	Total bytes utilized in the forward direction for headers
44	Fwd Pkt Len Mean	The mean deviation of the size of the packet in the forward direction
50	Fwd Seg Size Avg	Average size observed in the forward direction
74	Tot Fwd Pkts	Total packets in the forward direction
76	TotLen Fwd Pkts	Total size of packet in the forward direction

**Table 7:** Feature subsets selected based on the XRDI method.

FS Method	Dataset	Sub-Features Selected
XRDI	InSDN	8, 19, 28, 29, 30, 33, 37, 48, 65, 74
	CICIDS2017	8, 15, 37, 43, 44, 50, 58, 70, 75, 76
	CICIDS2018	37, 40, 44, 46, 50, 57, 71, 73, 74, 76

**Table 8:** Experimental environment.

Operating System	Windows 10 64-Bit
Memory	24 GB
CPU	Intel (R) Core (TM) i5-8250U CPU @ 1.60 GHz (Santa Clara, CA, USA)
Win-Python	3.11.5
Scikit-learn	1.3.1
Ubuntu	16.04 LTS
Ryu	4.34
Mininet	2.3.1b4
Ubuntu-Python	3.7.1

**Table 9:** Accuracy on three datasets using different FS methods.

FS Method _FS Number	Model	InSDN				CICIDS2017				CICIDS2018			
		Acc	Pre	Rec	F1	Acc	Pre	Rec	F1	Acc	Pre	Rec	F1
ALL_77	DT	99.98	99.99	99.98	99.98	99.97	99.97	99.97	99.97	100	100	100	100
	SVM	99.95	99.95	99.97	99.96	99.86	99.83	99.89	99.86	99.99	100	99.99	99.99
	RF	99.99	100	99.98	99.99	99.98	100	99.97	99.98	100	100	100	100
	LR	99.96	99.94	99.98	99.96	99.86	99.84	99.87	99.86	99.99	99.99	100	99.99
XGBoost_10	DT	99.97	99.99	99.96	99.97	99.97	99.98	99.97	99.97	99.99	99.99	100	99.99
	SVM	99.9	99.91	99.91	99.91	89.67	87.63	92.49	89.94	99.99	99.99	99.99	99.99
	RF	99.98	99.99	99.96	99.98	99.99	99.99	99.99	99.99	100	100	100	100
	LR	99.86	99.94	99.8	99.87	89.57	82.86	99.87	90.57	99.99	99.98	99.99	99.99
RF_10	DT	99.98	99.99	99.97	99.98	99.93	99.99	99.88	99.93	99.99	99.98	100	99.99
	SVM	99.51	99.53	99.58	99.56	98.72	97.79	99.7	98.74	99.95	99.91	99.99	99.95
	RF	99.98	99.99	99.97	99.98	99.93	99.98	99.88	99.93	99.99	99.98	100	99.99
	LR	99.51	99.53	99.58	99.55	95.17	91.29	99.91	95.4	99.96	99.92	99.99	99.96
DT_10	DT	99.98	99.99	99.98	99.99	99.96	99.96	99.97	99.96	100	100	100	100
	SVM	99.94	99.96	99.92	99.94	97.58	98.89	96.25	97.56	99.99	100	99.99	99.99
	RF	99.99	100	99.98	99.99	99.99	99.99	99.99	99.99	100	100	100	100
	LR	99.93	99.94	99.94	99.94	88.86	88.38	89.58	88.97	99.99	99.98	99.99	99.99
IG_10	DT	99.97	99.98	99.96	99.97	99.96	99.98	99.93	99.96	99.99	100	99.99	99.99
	SVM	99.92	99.95	99.9	99.93	98.79	97.84	99.8	98.81	99.99	99.99	99.99	99.99
	RF	99.97	100	99.95	99.97	99.96	99.98	99.94	99.96	99.99	100	99.99	99.99
	LR	99.84	99.92	99.78	99.85	98.75	97.78	99.78	98.77	99.96	99.93	100	99.96
XRDI_10	DT	99.97	99.99	99.96	99.98	99.95	99.97	99.94	99.95	99.99	100	99.99	99.99
	SVM	99.89	99.89	99.91	99.9	99.05	98.31	99.82	99.06	99.99	99.98	99.99	99.99
	RF	99.97	99.99	99.96	99.98	99.96	99.98	99.94	99.96	99.99	100	99.99	99.99
	LR	99.71	99.9	99.58	99.74	99.15	98.49	99.83	99.16	99.96	99.93	100	99.96

**Table 10:** Results of ten DDoS attack detection experiments on the simulation platform.

No.	Detecting Time (ms)	Predict Time (ms)
1	658.542	0.225
2	247.587	0.297
3	294.977	0.224
4	611.385	0.217
5	255.618	0.194
6	257.66	0.203
7	214.334	0.269
8	247.119	0.202
9	485.628	0.212
10	254.248	0.261

**Table 11:** Comparison with existing studies.

Literature	Dataset	FS Method	FS Number	Model	Acc	Pre	Rec	F1	Predict Time (ms)
[33]	NSL-KDD	Information gain	12	DT	97.2				0.3
[37]	KDDCup99	Stacked-based FS	11	XGBoost	99.87				
[23]	TOR	Boruta	15	KNN	94–96				
[24]	CICIDS2017	chi-rev	40	SVM	92.73	89.34	87.12	88.17	
				DT	99.88	99.8	99.84	99.82	
				NB	87.06	84.89	70.71	74.74	
				RF	99.9	99.8	99.89	99.85	
[10]	Honeybird	LD-KPCA	15	SVM	95.37				
[36]	KDDCup99	CFS, BFS	11	FURIA	99.58				
[34]	CICIDS2018	The binary grey wolf optimization algorithm	26	RF	99.13	98.43	99.92	99.13	
Proposed	InSDN	XRDI	10	DT	99.97	99.99	99.96	99.98	0.2304
	CICIDS2017				99.95	99.97	99.94	99.95	
	CICIDS2018				99.99	100	99.99	99.99	

## Data Availability

The InSDN dataset is available at https://aseados.ucd.ie/datasets/SDN/ (accessed on 9 January 2024). The CICIDS2017 dataset is available at https://www.unb.ca/cic/datasets/ids-2017.html (accessed on 24 December 2023). The CICIDS2018 dataset is available at https://www.unb.ca/cic/datasets/ids-2018.html (accessed on 15 March 2024).
